# Automatic Removal of Physiological Artifacts in OPM-MEG: A Framework of Channel Attention Mechanism Based on Magnetic Reference Signal

**DOI:** 10.3390/bios15100680

**Published:** 2025-10-09

**Authors:** Yong Li, Dawei Wang, Hao Lu, Yuyu Ma, Chunhui Wang, Binyi Su, Jianzhi Yang, Fuzhi Cao, Xiaolin Ning

**Affiliations:** 1 Key Laboratory of Ultra-Weak Magnetic Field Measurement Technology, Ministry of Education, School of Instrumentation and Optoelectronic Engineering, Beihang University, 37 Xueyuan Rd., Haidian District, Beijing 100083, China; 2 Hangzhou Institute of National Extremely-Weak Magnetic Field Infrastructure, 465 Binan Rd., Binjiang District, Hangzhou 310000, China; 3 Beijing Microelectronics Technology Institute, Beijing 100076, China; 4 School of Artificial Intelligence and Data Science, Hebei University of Technology, Tianjin 300130, China; 5 Hefei National Laboratory, Gaoxin District, Hefei 230088, China; 6 Shandong Key Laboratory for Magnetic Field-Free Medicine & Functional Imaging, Institute of Magnetic Field-Free Medicine & Functional Imaging, Shandong University, 27 South Shanda Rd., Licheng District, Jinan 250100, China

**Keywords:** optically pumped magnetometer, physiological artifacts, randomized dependence coefficient, channel attention mechanism, magnetic reference signal, event-related field, signal-to-noise ratio

## Abstract

The high spatiotemporal resolution of optically pumped magnetometers (OPMs) makes them an essential tool for functional brain imaging, enabling accurate recordings of neuronal activity. However, physiological signals such as eye blinks and cardiac activity overlap with neural magnetic signals in the frequency domain, resulting in contamination and creating challenges for the observation of brain activity and the study of neurological disorders. To address this problem, an automatic physiological artifact removal method based on OPM magnetic reference signals and a channel attention mechanism is proposed. The randomized dependence coefficient (RDC) is employed to evaluate the correlation between independent components and reference signals, enabling reliable recognition of artifact components and the construction of training and testing datasets. A channel attention mechanism is subsequently introduced, which fuses features from global average pooling (GAP) and global max pooling (GMP) layers through convolution to establish a data-driven automatic recognition model. The backbone network is further optimized to enhance performance. Experimental results demonstrate a strong correlation between the magnetic reference signals and artifact components, confirming the reliability of magnetic signals as artifact references for OPM-MEG. The proposed model achieves an artifact recognition accuracy of 98.52% and a macro-average score of 98.15%. After artifact removal, both the event-related field (ERF) responses and the signal-to-noise ratio (SNR) are significantly improved. Leveraging the flexible and modular characteristics of OPM-MEG, this study introduces an artifact recognition framework that integrates magnetic reference signals with an attention mechanism. This approach enables highly accurate automatic recognition and removal of OPM-MEG artifacts, paving the way for real-time, automated data analysis in both scientific research and clinical applications.

## 1. Introduction

Optically pumped magnetometers (OPMs) based on the spin-exchange relaxation-free (SERF) principle offer ultra-high sensitivity for measuring extremely weak magnetic fields [1], substantially enhancing the performance of modular, detachable magnetoencephalography systems (OPM-MEG) assembled with such sensors. Compared with conventional superconducting quantum interference device (SQUID)-based MEG, OPM-MEG does not require an ultra-low-temperature environment (−270 °C) and can operate under normal room-temperature conditions [2]. In addition, OPM-MEG provides advantages such as wearability and closer proximity to the scalp [3]. Furthermore, it is more cost-effective than SQUID-MEG, making it more accessible to researchers and clinicians [4], thereby facilitating broader adoption of MEG in both research and clinical applications.

Magnetoencephalography (MEG) acquires brain signals by measuring the extracranial magnetic fields generated by neuronal activity [5]. One of its key advantages is that magnetic field propagation is minimally affected by the conductive and structural properties of the head, allowing the recorded data to retain a greater proportion of the brain’s original signal content [6]. However, the measured signals often contain substantial physiological artifacts originating from other parts of the body, such as those produced by eye blinks and cardiac activity [7]. These artifacts are transmitted to the head and share the same frequency bands (1–20 Hz) as conventional neuronal sources, including δ (1–3Hz), θ (4–7Hz), and α (8–13Hz) rhythms, thereby confounding the target brain signals within this range [8]. This overlap poses a significant challenge for extracting the desired neural information [9]. Consequently, for both conventional SQUID-MEG and the newer OPM-MEG, accurate recognition and removal of such physiological artifact interference remain primary prerequisites for reliable brain signal analysis [10]. A distinctive complication for OPM-MEG, however, lies in the flexible sensor placement afforded by its design [11], which increases the complexity of artifact detection.

In conventional magnetoencephalography (MEG), identifying and removing physiological artifacts caused by eye blinks and cardiac activity typically relies on independent component analysis (ICA) combined with manual inspection or external reference channels [12]. Because ICA decomposes neural signals and noise in a random order, manual methods require expert judgment to identify and remove artifact-related components, making the process both time-consuming and unsuitable for real-time analysis [13]. To enhance automation, electrooculography (EOG) and electrocardiography (ECG) are often recorded as references for identifying and removing corresponding components [9]. Within this framework, several automated approaches have been proposed. For example, Sun et al. developed a resampled moving average subtraction (RMAS) method based on ECG references to achieve semi-automatic removal of cardiac artifacts through averaging of heartbeat waveforms [14]. Hasasneh et al. trained a deep convolutional neural network (DCNN) on EOG- and ECG-labeled artifact data to enable fully automated classification and removal of ocular and cardiac artifacts [15]. However, these approaches increase the complexity of data acquisition and may cause discomfort or introduce additional electromyographic artifacts in participants. Moreover, due to differences in measurement principles between magnetic and electrical signals, the effectiveness of electrical references for correcting MEG artifacts is limited [13,16]. Most existing methods are also designed for fixed SQUID-MEG sensor layouts, and their direct application to the flexible sensor arrangements of OPM-MEG may result in bias. Given the modular and detachable nature of OPM-MEG, automated removal of artifacts based on magnetic reference signals becomes possible. Nevertheless, no prior study has utilized magnetic sensor signals as references to automatically identify and remove physiological artifacts such as eye blinks and cardiac activity, and the feasibility of this approach remains to be demonstrated.

Accurate and automated recognition and removal of physiological artifact components based on magnetic sensor reference signals requires two essential steps. First, the feasibility of using magnetic sensor signals as reference signals must be established, as this directly determines the accuracy of the data used in the automatic recognition stage. In conventional approaches employing electrical reference signals, artifact components are recognized by measuring their correlation with the reference signals. For example, Lukas et al. recognized blink artifacts using the Pearson correlation between artifacts and electrical reference signals, which effectively detects linearly correlated components [9]. This principle can be extended by evaluating the correlation between magnetic reference signals and artifact components. However, ICA-separated artifact components are not strictly linearly related to their sources. To address this, the present study employs the randomized dependence coefficient (RDC) to capture both linear and nonlinear dependencies across arbitrary dimensions, thereby improving the detection of mixed or nonlinear artifact components. Second, an efficient classification algorithm is required for automated artifact recognition. Recent advances in neural networks highlight the attention mechanism, which, akin to the human visual system, selectively focuses on the most informative features while suppressing irrelevant details [17]. In convolutional neural networks (CNNs), the channel attention mechanism plays a key role by assigning saliency weights to output feature map channels, thus enhancing feature discrimination. Integrating this mechanism into the automatic recognition of OPM-MEG physiological artifacts—particularly for time sequence features—can substantially improve recognition accuracy [18].

This study first employed two OPM sensors to respectively record ocular and cardiac magnetic signals, which served as magnetic reference signals for physiological artifacts. Subsequently, the RDC was used to assess the correlation between independent components and the reference signals, thereby validating the feasibility of artifact recognition based on magnetic references. Based on a correlation threshold, a dataset comprising three classes—blink artifacts, cardiac artifacts, and non-artifact components—was constructed for training and testing. An automatic artifact recognition model incorporating a channel attention mechanism was then developed. This mechanism weighted feature maps by integrating features from global average pooling (GAP) and global max pooling (GMP) layers, enhancing the representation of physiological artifact-related features while suppressing those associated with neuronal activity, thus preserving features critical for artifact recognition. The effectiveness of the channel attention mechanism in capturing temporal features of physiological artifacts in OPM-MEG data was validated through ablation experiments across multiple backbone networks. leading to the selection of optimal backbone architecture to improve model performance. Finally, artifact components were removed based on automatically recognized component indices, and the effectiveness of artifact removal was evaluated both qualitatively and quantitatively using event-related field (ERF) waveforms and signal-to-noise ratio (SNR) metrics.

## 2. Materials and Methods

The correlation between independent components and their corresponding artifact signal sources can serve as a preliminary basis for artifact recognition. In this study, we employ the RDC method to quantify the correlation between independent components and magnetic reference signals. However, relying solely on RDC is insufficient for fully automated artifact identification: threshold-based selection requires manual parameter tuning and is sensitive to inter-subject variability, task paradigms, and background noise, which can lead to missed detections or false positives. To overcome these limitations, we integrate RDC with a deep learning-based classifier, allowing the model to leverage physiologically meaningful correlation information while learning complex, nonlinear patterns for robust, parameter-free artifact recognition. Once artifact components are recognized by the model, they are subsequently removed using the ICA procedure, ensuring that physiologically irrelevant signals are effectively excluded. The following methodology section sequentially describes the construction of the proposed automatic artifact recognition framework, including participants and stimulation paradigm, data acquisition, correlation measurement with magnetic reference signals, dataset construction, and model training.

### 2.1. Participants and Stimulation Paradigm

This study involved 16 healthy volunteers, consisting of 14 males and 2 females, aged between 24 and 30 years. All participants were right-handed native Mandarin speakers with no known history of congenital developmental disorders, auditory impairments, neurological conditions, or psychiatric disorders. Each participant provided informed consent and voluntarily took part in the study. The research protocol received approval from the Ethics Committee of Beihang University and adhered to the ethical principles outlined in the Declaration of Helsinki.

**Figure 1:**
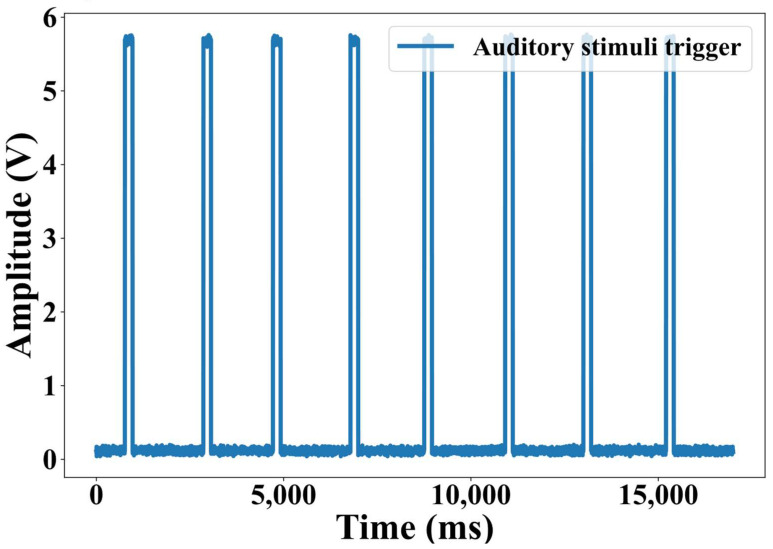
Waveform of partial auditory stimulus trigger signal.

In this study, a beep auditory stimulation paradigm [19] was employed to conduct an auditory experiment and collect data to validate the effectiveness of the proposed method. The auditory stimuli were generated using Psychtoolbox, output via the audio port of the experimental host computer and delivered to the participants’ ears through non-magnetic silicone tubing. The stimuli consisted of standard 1 kHz pure tones, each lasting 200–300 ms, with an inter-stimulus interval of 2 s. The experimental session comprised a total of 800 trials. A representative trigger waveform of the auditory stimuli is shown in Figure 1.

### 2.2. Data Acquisition and Preprocessing

**Figure 2:**
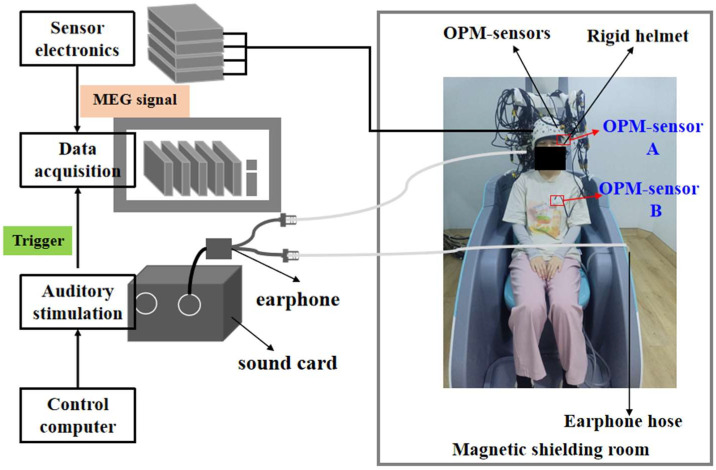
Schematic diagram of the data acquisition system.

The auditory experimental data were collected using the OPM-MEG system shown in Figure 2. This system employed 32 s-generation optically pumped magnetometers (Quspin Inc., Louisville, CO, USA) to measure radial neuromagnetic signals. Simultaneously, two additional OPM sensors were used to record ocular and cardiac magnetic signals as artifact reference signals. A PXI-based computer (PXIC-7318C, ART Technology Inc., Beijing, China) served as the acquisition unit to record all system signals. The experimental setup operated inside a magnetically shielded room (MSR) with a residual magnetic field amplitude of less than 10 nT.

After signal acquisition, all data were band-pass filtered to the range of 1.5–40 Hz, segmented into 10 s epochs, and then processed using the fast independent component analysis (FastICA) algorithm [20] to achieve component separation of the OPM-MEG signals. The OPM sensors were sampled at 1000 Hz, resulting in 10,000 discrete points per epoch. Since blink artifacts do not exhibit strict periodicity, and to fully demonstrate the feasibility of using magnetic reference signals while ensuring diversity in the subsequently obtained blink data, the study acquired MEG and magnetic reference signals not only during natural blinking, where participants sat comfortably and blinked spontaneously without any specific task requirements, but also during rapid blinking, where participants were explicitly instructed to blink at a faster pace (approximately 1–3 blinks per second).

Subsequent to data acquisition and preprocessing, data from 12 participants were utilized to establish the training and testing datasets for the physiological artifact automatic recognition model, with the remaining 4 participants’ data reserved for evaluating the effectiveness of artifact removal.

### 2.3. Correlation Measurement of Magnetic Reference Signals

In order to quantitatively demonstrate the feasibility of magnetic reference signals, we use the RDC [21] to measure the feature correlation between the ICA separation component and the magnetic reference signal. The process is as follows: first, the reference signal, such as the blink magnetic reference signal X, and a component signal Y of the ICA-derived are used as inputs. A copula transform is then applied to both signals to homogenize the samples to the (0,1) interval and remove the influence of marginal distributions.

(1) $$\left\{\begin{array}{l} {\tilde{X}}^{\left(m\right)}=F_{n}(X^{\left(m\right)})\\ {\tilde{Y}}^{\left(m\right)}=F_{n}(Y^{\left(m\right)}) \end{array}\right.$$

where m represents the m-th sample point, and F is the empirical distribution function of Copula transform, which is defined as follows:

(2) $$F_{n}(x)=\frac{1}{n+1}\sum_{j=1}^{n}1\left\{X^{\left(j\right)}\leq x\right\}$$

where x corresponds to any specific sample value in the signal, $X^{\left(j\right)}$ denotes the j-th observation in the dataset, and n is the total number of samples. The symbol $1\left\{X^{\left(j\right)}\leq x\right\}$ represents the indicator function, which equals 1 if the j-th sample is not greater than x, and 0 otherwise. Accordingly, the summation counts the number of samples less than or equal to x, and dividing this by n+1 maps each raw sample onto the interval (0,1). The use of n+1 instead of n ensures that the mapped values do not take boundary points 1, thereby maintaining numerical stability.

Then, the high-dimensional features are constructed by random projection and activation function in Copula space, and the specific operation is as follows:

(3) $$\left\{\begin{array}{l} \phi (X)=\sin(\tilde{X}W_{X}+b_{X})\in R^{n\times k}\\ \phi (Y)=\sin(\tilde{Y}W_{Y}+b_{Y})\in R^{n\times k} \end{array}\right.$$

where $W_{X},W_{Y}\in R^{1\times k}$ are random weight matrix, which is obtained by random sampling from normal distribution $Ν\left(0,s^{2}\right)$. Parameter s controls the projection scale, and the default setting is 1/6. k is the number of random projections, and the default setting is 20. $b_{X},b_{Y}\in R^{k}$ are random bias term, which is obtained by random sampling in the $\mathrm{Uniform}\left(0,2\pi \right)$ distribution.

Finally, by performing regularized canonical correlation analysis (RCCA) [22] on the two groups of features ϕ(X) and ϕ(Y) after projection, RDC is obtained to measure the correlation between the ICA component and the magnetic reference signal component, as follows:

(4) $$\mathrm{RDC}(X,Y)=\underset{a,b}{\max}\frac{\mathrm{cov}(a^{Τ}\phi (X),b^{Τ}\phi (Y))}{\sqrt{\mathrm{var}(a^{Τ}\phi (X))\mathrm{var}(b^{Τ}\phi (Y))}}$$

where $a,b\in R^{k}$ are the projection direction vectors, and solving the problem is equivalent to maximizing the correlation coefficient of the two vectors in the projection space.

### 2.4. Dataset Construction Method

To enable automatic artifact recognition, the dataset in this study was constructed based on thresholding the correlation scores measured using the RDC method. Specifically, blink artifact components and cardiac artifact components were defined as ICA components exhibiting the highest correlation with their corresponding magnetic reference signals and exceeding a predefined correlation threshold δ. In contrast, non-artifact components were defined as ICA components with correlation values below a separate threshold λ. The threshold parameters δ and λ were manually determined according to the characteristics of the acquired data.

Blink and cardiac artifact components were used as positive samples, whereas non-artifact components were used as negative samples. To balance the distribution of positive and negative samples during model training, the ratio of positive to negative samples was maintained at 1:2, corresponding to a blink artifact: cardiac artifact: non-artifact ratio of 1:1:4. Since the polarity of ICA component amplitudes is inherently random, all samples were inverted to generate additional samples during dataset construction, thereby increasing the dataset size and enhancing the generalization capability of the automatic artifact recognition model.

### 2.5. Model Construction

After verifying the feasibility of the magnetic reference signal, it is necessary to establish an effective model for recognition and remove artifacts automatically. This paper refers to a new complementary attention network (CAN) designed by Su et al. [23]. Their research adaptively suppressed the background noise features of two-dimensional solar cell electroluminescent (EL) images. It highlighted the defect features by connecting the new channel attention sub-network and the spatial attention sub-network in turn, which objectively proved that GAP and GMP could collect distinctive features for object recognition. Since the OPM-MEG signal artifacts recognition input is a one-dimensional time sequence signal, it is of little significance to consider the impact of the spatial attention mechanism. Therefore, in this paper, we only focus on introducing the attention mechanism of channels into the construction of the OPM-MEG signal artifacts time sequence features recognition network framework, CA-SeqNet, which focuses on selecting channels containing expected information.

**Figure 3:**
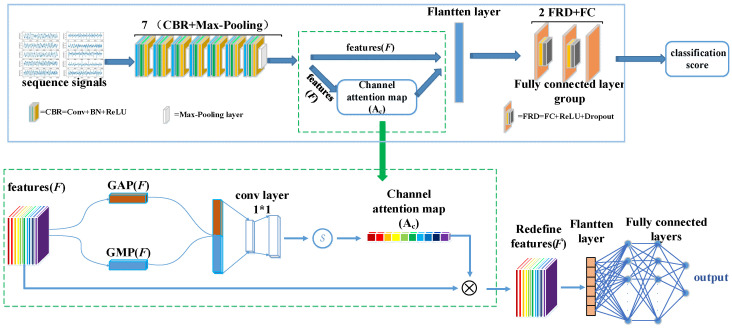
Network architecture of CA-SeqNet. The backbone consists of 7 (CBR + Max-Pooling) blocks, where CBR = Conv1d + BatchNorm1d + ReLU. The channel attention module is constructed from the following components: a combination of GAP and GMP, a 1×1 convolutional layer, and a sigmoid activation layer. The fully connected layer group consists of two FRD modules, each comprising a fully connected layer (FC), a ReLU activation layer, and a Dropout layer, followed by a final FC layer.

The network architecture of CA-SeqNet is shown in Figure 3. First, the input signal is processed by the backbone network to extract intermediate feature maps F. The backbone network is composed of seven repeated units, each consisting of a CBR module followed by a Max-Pooling layer, with each CBR module comprising a one-dimensional convolution (Conv1D), batch normalization (BatchNorm1D), and a ReLU activation function in sequence. Then, the channel attention module performs weighted enhancement of the channel features in F to obtain the optimized feature $F^{\prime }$. Next, two FRD modules—each consisting of a fully connected layer, ReLU activation, and dropout—further select useful information from $F^{\prime }$. Finally, a fully connected layer completes the classification of artifact categories. Specifically, the channel attention module first applies global average pooling (GAP) and global max pooling (GMP) to the feature F, yielding two channel descriptors, GAP(F) and GMP(F). These are then concatenated as $G=[\mathrm{GAP}(F),\mathrm{GMP}(F)]\in R^{1\times 1\times 2C}$ to integrate global statistical and peak response information. Subsequently, G is linearly projected and fused through a 1×1 convolution, mapping it back to the same number of channels as the original feature map, and a Sigmoid activation generates the channel attention map $A_{c}$. Finally, $A_{c}$ is multiplied with the original feature F channel-wise to obtain the enhanced feature $F^{\prime }$, achieving channel-wise feature attention enhancement. This design enables the network to adaptively emphasize channels relevant to artifact recognition while suppressing redundant or noisy channels, thereby enhancing overall recognition performance.

**Figure 4:**
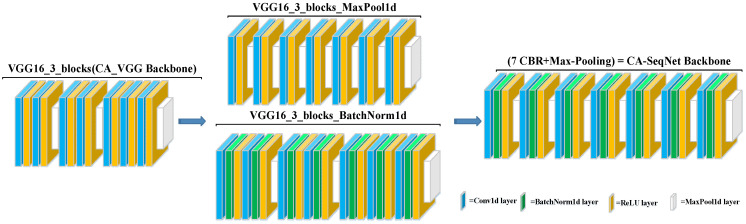
Structural evolution of the backbone. The baseline VGG16_3_blocks is constructed from the first three blocks of VGG16. VGG16_3_blocks_BatchNorm1d adds a BatchNorm1d after each Conv1d, and VGG16_3_blocks_MaxPool1d adds a MaxPool1d after each ReLU unless a MaxPool1d is already present. Combining both methods yields the final backbone of 7 (CBR + Max-Pooling) blocks (CA-SeqNet Backbone), where CBR = Conv1d + BatchNorm1d + ReLU.

The backbone of CA-SeqNet was obtained by selecting the first three blocks of the VGG16 network [24] as the main branch, serving as the baseline architecture for further evolution, as shown in Figure 4. Based on this baseline, we investigated two independent backbone optimization strategies: (i) adding a BatchNorm1d layer after each Conv1d layer to normalize intermediate activations, thereby improving training stability, mitigating gradient vanishing, and accelerating convergence; and (ii) inserting a MaxPool1d layer after each ReLU activation function to emphasize salient temporal features, reduce temporal length, and enhance robust-ness to noise, but if a MaxPool1d layer already follows the ReLU, no additional layer is added to avoid repeated pooling. Ablation studies indicated that these two strategies are complementary. Therefore, we combined both modifications to construct the final backbone network, composed of seven repeated units of a CBR module followed by a Max-Pooling layer (CBR + Max-Pooling). Each CBR module consists of a Conv1d layer followed by BatchNorm1d and a ReLU activation. This final architecture achieved higher accuracy compared with networks employing either modification alone. All the above neural network operations, including Conv1d, BatchNorm1d, ReLU, MaxPool1d, and others, were implemented within the PyTorch framework (version 2.4.1) [25].

The CA-SeqNet network requires labeled input signal sequences to learn the corresponding signal features. Therefore, the input signal sequences must undergo labeling, with the label sequence $\left\{x_{n}(\delta )\right\}$ expressed as:

(5) $$\left\{x_{n}(\delta )\right\}=\left\{x_{1}(\delta ),x_{2}(\delta ),x_{3}(\delta ),x_{4}(\delta ),\cdots ,x_{n}(\delta )\right\}$$

where $x_{n}(\delta )$ denotes the n-th data sample fed into the network, and its value is determined according to Equation (6):

(6) $$x_{n}(\delta )=\left\{\begin{array}{l} 0,\delta =\mathrm{Cardiac}\_\mathrm{Artifact}\\ 1,\delta =\mathrm{Blink}\_\mathrm{Artifact}\\ 2,\delta =\mathrm{None}\_\mathrm{Artifact} \end{array}\right\}$$

where δ represents the artifact class label, while Cardiac_Artifact, Blink_Artifact, and None_Artifact correspond to the classes of cardiac artifact, blink artifact, and non-artifact components, respectively.

The prediction result of the model and the real label loss are calculated by using the multi-classification cross-entropy loss function [26], and its expression is:

(7) $$Loss=-\sum_{i=1}^{N-1}y_{i}\log(p_{i})$$

where $p=\left\{p_{0},p_{1},p_{2},\cdots ,p_{N-1}\right\}$ is the probability distribution of model output, $y=\left\{y_{0},y_{1},y_{2},\cdots ,y_{N-1}\right\}$ is the true probability distribution of sample labels, and N is the number of sample labels.

During the training process, each epoch outputs loss once to monitor the learning effect of the model. The parameter optimizer uses the adaptive moment estimation optimizer [27]. The instantiation of the network structure involves inputting the training set into the model and calculating the training loss of the current batch. Then the existing gradient is cleared, the error is propagated back, and the network parameters are updated to complete the training process.

## 3. Experiments and Results

To verify the reliability of artifact recognition based on magnetic reference signals and to achieve automatic detection and removal of physiological artifacts, the OPM-MEG signals in this study were first decomposed into components using the FastICA method. The correlation between the reference signals and the separated components was then calculated using the RDC method. Based on correlation thresholds, training and testing datasets for the automatic recognition model were constructed. Subsequently, multiple network backbones were employed for comparative and ablation experiments, and the performance of the channel-attention convolutional network module was evaluated in the artifact time sequence features recognition task. Finally, once blink and cardiac artifact components were recognized by the CA-SeqNet framework, they were subsequently removed through the ICA procedure, and the effectiveness of the proposed network was assessed through ERF [28] waveform and signal-to-noise ratio analyses based on the MEG signals.

### 3.1. Correlation Analysis Results

**Figure 5:**
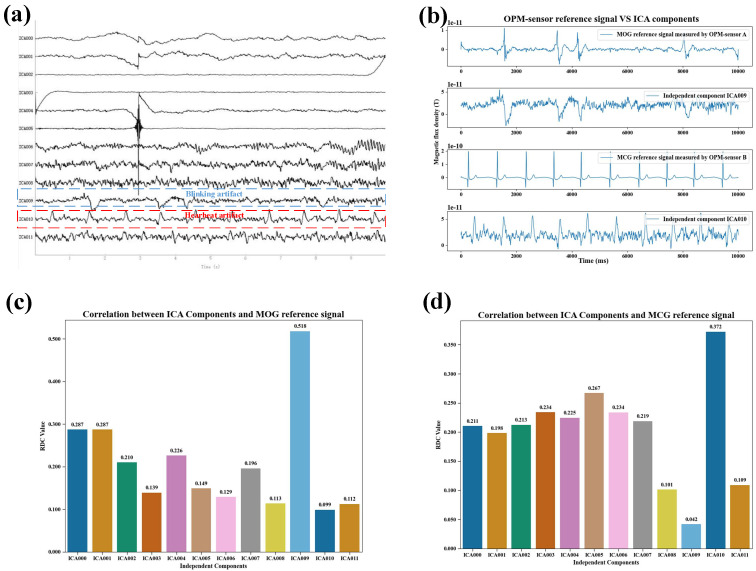
Feasibility verification of the magnetic reference signal in normal blinking. (**a**) ICA-derived components of the subject’s signals under normal blinking conditions; (**b**) Qualitative comparison of magnetic reference signals and ICA-derived artifact components under normal blinking conditions; (**c**) Correlation of MOG signals with ICA-derived components under normal blinking conditions; (**d**) Correlation of MCG signals with ICA-derived components under normal blinking conditions.

Figure 5 presents an example of the correlation results between the magnetic reference signals and the components separated by the FastICA algorithm during normal blinking. Figure 5a shows the neuromagnetic signal components obtained via FastICA decomposition under normal blinking conditions. Figure 5b provides a qualitative comparison between the magnetic reference signal and the ICA-separated artifact components in the same condition. The blink magnetic reference (MOG) signal recorded by OPM sensor A exhibits non-periodic peak variations, similar to those observed in the ICA-derived blink artifact component. The cardiac magnetic reference (MCG) signal recorded by OPM sensor B displays the typical QRS complex pattern [29] and contains repeated peaks with a periodicity comparable to that of the ICA-derived cardiac artifact component. Figure 5c illustrates the correlation between the MOG signal and the ICA components under normal blinking. The correlation with the ICA blink artifact component is the highest and is markedly greater than that with other components. Figure 5d shows the correlation between the MCG reference signal and the ICA components, where the correlation with the ICA cardiac artifact component is the highest and significantly exceeds that with other components.

**Figure 6:**
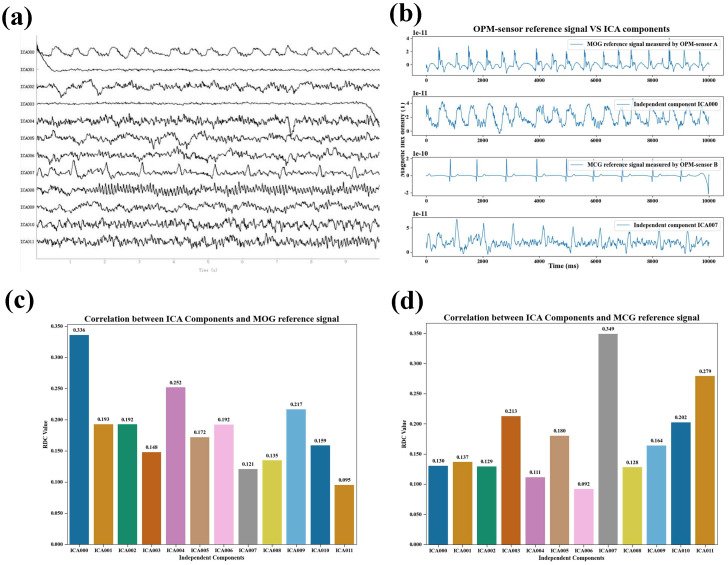
Feasibility verification of the magnetic reference signal in fast blinking. (**a**) ICA-derived components of the subject’s signals under fast blinking conditions; (**b**) Qualitative comparison of magnetic reference signals and ICA-derived artifact components under fast blinking conditions; (**c**) Correlation of MOG signals with ICA-derived components under fast blinking conditions; (**d**) Correlation of MCG signals with ICA-derived components under fast blinking conditions.

Figure 6 presents an example of the correlation results between the magnetic reference signals and the components separated by the FastICA algorithm during rapid blinking. Figure 6a shows the neuromagnetic signal components obtained through FastICA decomposition under rapid blinking conditions. Figure 6b provides a qualitative comparison between the magnetic reference signals and the ICA-separated artifact components in the same condition. It can be observed that the MOG signal recorded by OPM sensor A during rapid blinking exhibits rapid, non-periodic peak shifts, similar to the peak variations observed in the ICA-derived blink artifact component. The MCG signal recorded by OPM sensor B still displays the typical QRS complex pattern and contains repeated peaks with periodicity comparable to that of the ICA-derived cardiac artifact component. Figure 6c illustrates the correlation between the MOG signal and the ICA components under rapid blinking, again showing the highest correlation with the ICA blink artifact component, which is significantly greater than that with any other component. Figure 6d presents the correlation between the MCG signal and the ICA components under rapid blinking, likewise showing the highest correlation with the ICA cardiac artifact component, markedly exceeding the correlations with all other components.

The above correlation analysis results demonstrate that the magnetic reference signals exhibit a significant correlation with the artifact component signals, indicating that they can serve as a reliable basis for the recognition of ICA-derived artifact components that require removal.

### 3.2. Dataset Construction Results

**Figure 7:**
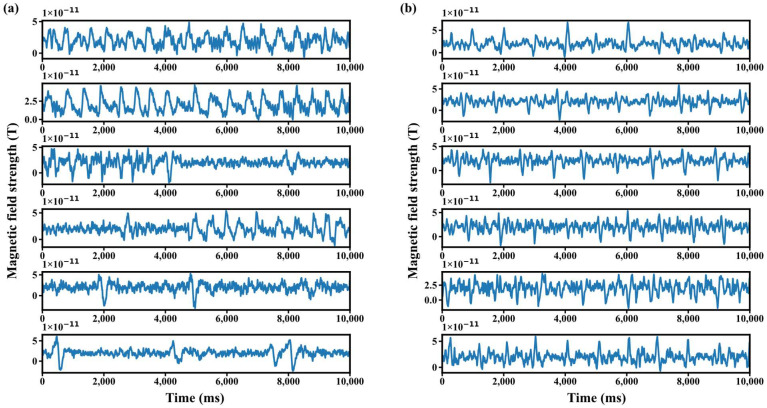
Artifact effects in the constructed dataset. (**a**) blink artifacts; (**b**) heartbeat artifacts.

During dataset construction, the threshold parameters δ and λ were set to 0.3 and 0.2, respectively. The resulting dataset is illustrated in Figure 7. As shown, blink artifacts exhibit diverse patterns but relatively stable amplitudes, while cardiac artifacts present repeated peaks with a periodicity similar to that of the MCG signal, consistently ranging between 900 and 1000 ms. After inverting the original samples, the total number of positive and negative samples in the new dataset reached 5760, representing a 50% increase compared to the original dataset. The detailed sample statistics of the time sequence signal dataset are summarized in Table 1.

### 3.3. Model Training Details and Evaluation Indicators

In order to achieve the real-time automatic removal of physiological artifacts, this paper trains an automatic artifact recognition network model based on the constructed dataset, enabling automatic recognition without reference signals. The data were split into training and testing sets at a ratio of 4:1. To ensure that the model adequately learned the characteristics of the samples, the number of training epochs was set to 100, with a batch size of 16. Training was conducted on an NVIDIA A100 40GB PCIE GPU. To maintain the stability of training results, a fixed random seed of 42 was used to reduce the randomness in neural network weight initialization. Since variations in optimizer parameters can also affect training stability, the initial learning rate was set to 0.001 and adjusted using a learning rate decay strategy to stabilize training outcomes further. Specifically, a decay factor of 0.1 was applied, reducing the learning rate by a factor of 0.1 every 10 epochs.

Time sequence features recognition of physiological artifacts belongs to a multi-classification problem. There are four basic concepts in the evaluation method of classification problems [30], as shown in Table 2.

The commonly used evaluation indices of multi-classification problems include: Precision (PR), Recall (RE), Accuracy (ACC), macro-averaged F1 score (Macro_F1), etc.

The precision rate represents the proportion of samples with positive predictions and correct prediction results among all samples with positive predictions, and its expression is:

(8) $$\mathrm{Precision}=\frac{\mathrm{TP}}{\mathrm{TP}+\mathrm{FP}}$$

The recall rate represents the proportion of samples with positive prediction and correct prediction results to all the actual non-positive samples, and its expression is:

(9) $$\mathrm{Recall}=\frac{\mathrm{TP}}{\mathrm{TP}+\mathrm{FN}}$$

The accuracy rate represents the proportion of all samples with correct prediction results to all samples, and its expression is:

(10) $$\mathrm{Accuracy}=\frac{\mathrm{TP}+\mathrm{TN}}{\mathrm{TP}+\mathrm{FN}+\mathrm{FP}+\mathrm{FN}}$$

The F1_score is usually used to measure binary classification model, and its expression is Formula (10), which can be regarded as the weighted average of precision rate and recall rate. The Macro_F1 is commonly used to evaluate multi-class models, as it primarily measures the balanced performance of the model across all classes, and its expression is shown in Equation (12):

(11) $$F_{1}\_\mathrm{Score}=\frac{2*\mathrm{Precision}*\mathrm{Recall}}{\mathrm{Precision}+\mathrm{Recall}}$$

(12) $$\mathrm{Macro}\_F_{1}=\frac{1}{n}\sum_{i=1}^{n}F_{1i}\times 100\%$$

where n represents the total number of classification categories.

### 3.4. Ablation Experiment

#### 3.4.1. Ablation Study on the Attention Mechanism

In order to prove the effectiveness of the channel attention mechanism introduced in this paper, we use the long short-term memory network [31] (LSTM), gated unit network [32] (GRU), AlexNet neural network [33], FasterRCNN network [34], and DenseNet121 network [35], VGG16 [24] network and other common classification networks are used as network skeletons to train and predict the time sequence signal dataset of the artifacts to conduct model ablation experiments. The performance indicators obtained from the experiment are shown in Table 3, where networks prefixed with “CA” denote models augmented with the channel attention mechanism. The addition of channel attention sub-network (CA) module better promotes the classification results of the above-mentioned classical CNN model, which shows that the addition of CA module makes the network make full use of the distinguishing features of GAP and GMP layers, and proves that CA module can be widely used to improve the expressive ability of CNN architecture. It is worth noting that because our data is one-dimensional data and the pre-training data of the above model is two-dimensional, in order to make the model better adapt to the current task without causing data deviation, we do not use the pre-training model, but use our own training set for training throughout.

#### 3.4.2. Ablation Study on Backbone Architectures

Due to the limited amount of data, some networks failed to converge. In particular, even with the addition of the channel attention mechanism subnetwork module, VGG16 was unable to converge with a stable classification model. To address this problem, we conducted a backbone ablation experiment to explore the backbone architecture that is more suitable for this task, thereby establishing the final channel attention mechanism–based physiological artifact recognition network (CA-SeqNet) utilizing OPM-MEG magnetic reference signals. The performance metrics obtained from the experiments are summarized in Table 4, with the backbone architectures illustrated in Figure 3. As before, networks prefixed with “CA” denote models augmented with the channel attention mechanism. The CA-SeqNet model achieved an accuracy of 98.52% across the entire dataset, with recall and precision values of 98.36% and 97.96%, respectively, and a macro-F1 score of 98.15%. Compared with the baseline model using the basic VGG16_3_blocks as the backbone, all performance metrics were substantially improved. Furthermore, relative to the best-performing CA-DenseNet121 network reported in Table 3, the CA-SeqNet achieved improvements of 3.9%, 4.7%, 7.0%, and 6.0% in accuracy, precision, recall, and macro-F1 score, respectively.

In the ablation experiment for VGG_3_blocks, we found that all indexes of the network with attention mechanism were improved compared with the baseline model (VGG16_3_ blocks), and the accuracy, precision, recall and macro score of CA-SeqNet network were improved by 2.5%, 3.5%, 4.7% and 4.1%, respectively, compared with CA_(VGG16_3_blocks) network. However, adding the MaxPool1d layer or the BatchNorm1d layer to the backbone model alone will degrade the network performance compared to the CA-SeqNet. This may be because adding MaxPool1d will lose some detailed information, but combining with BatchNorm1d can stabilize the feature distribution and enhance the network’s learning ability. The effect of BatchNorm1d alone will not be perfect because the model is prone to underfitting due to the lack of down-sampling and information screening in the pool layer.

Table 5 presents the comparative performance metrics under stable conditions for cardiac artifacts, ocular artifacts, and non-artifacts. The final macro-averaged F1 score of 98.15% demonstrates that the model achieved a high level of performance in the multi-class classification task, exhibiting balanced effectiveness across all categories and avoiding dominance by the non-artifact class with its relatively larger sample size.

### 3.5. Artifact Removal Effect

**Figure 8:**
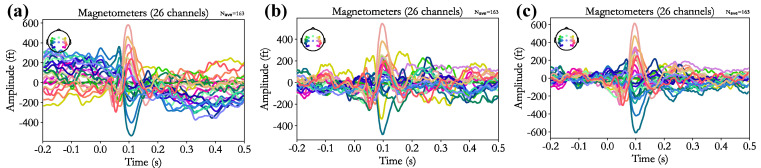
Wave diagram of event related field. (**a**) Original ERF; (**b**) ERF after manual removal artifacts; (**c**) ERF after SeqNet automatically removes artifacts.

Figure 8 presents representative event-related field (ERF) responses before and after artifact recognition and removal. Figure 8a shows the ERF waveform of a subject before artifact removal, where noise contamination results in abnormally elevated amplitudes both before and after stimulus onset, and the auditory M100 component is not distinguishable. Figure 8b illustrates the outcome following manual recognition and removal of ICA-derived artifacts, which reduces the overall ERF amplitude and effectively suppresses pre- and post-stimulus responses. Figure 8c depicts the results obtained with the proposed CA-SeqNet-based artifact recognition and removal approach. This method not only further attenuates pre- and post-stimulus amplitudes but also enhances the visibility of the auditory M100 component, yielding a more pronounced ERF response compared to manual artifact removal.

In order to quantitatively analyze the signal quality before and after artifact removal, we refer to the calculation method of signal-to-noise ratio (SNR) shown in reference [36] to measure the signal quality before and after artifact removal, as shown in Formula 13:

(13) $$\mathrm{SNR}=20\log_{10}\left(\frac{{\mathrm{RMS}}_{x_{poststim}}}{{\mathrm{RMS}}_{x_{prestim}}}\right)$$

where ${\mathrm{RMS}}_{x_{poststim}}$ represents the root mean square of 0–0.2s signal in the post-stimulation period, and ${\mathrm{RMS}}_{x_{prestim}}$ represents the root mean square of −0.2s–0s signal in the pre-stimulation period. We conducted a comparative analysis of artifact removal using data from four participants. Table 6 shows the results of comparing the proposed method of automatic recognition and removal of artifacts by CA-SeqNet network with the method of manual recognition and removal of artifacts. As shown, the signal-to-noise ratio (SNR) achieved by the proposed method consistently outperforms both manual artifact removal and the original data. These findings demonstrate the superior performance of the proposed approach in artifact recognition, thereby enhancing the overall quality of the data.

## 4. Conclusions and Discussion

Physiological artifacts such as ocular and cardiac signals have long posed a challenge in magnetoencephalography (MEG) recordings [37,38]. Conventional artifact recognition and removal methods often rely on correlation-based thresholds [39] or manual intervention [40]. Although widely adopted, these approaches are inherently limited by inter-subject variability and task-related differences, thereby constraining their reliability, reproducibility, and level of automation [41]. To address these challenges, the present study proposes a novel artifact removal framework designed explicitly for optically pumped magnetometer-based MEG (OPM-MEG). Leveraging the inherent flexibility and modularity of OPM systems, we introduce magnetic reference signals as an objective source of artifact components for the first time. Departing from conventional threshold-based strategies, the proposed approach constructs training and testing datasets based on objective correlation priors and integrates a channel attention mechanism to achieve automatic artifact recognition. This data-driven framework offers two key advantages: (i) reliable and fully automated detection of physiological artifacts, and (ii) reduced complexity of experimental procedures, underscoring the potential of OPM-MEG as a scalable tool for cognitive neuroscience and clinical research.

The ablation experiments on the attention mechanism demonstrate that the introduction of channel attention significantly enhances the classification performance of conventional CNN models. Building on this, the proposed CA-SeqNet not only achieves superior performance in classification tests but also yields favorable results in both qualitative and quantitative evaluations of event-related field waveforms and signal-to-noise ratio (SNR). In SNR analyses, artifact removal achieved through the CA-SeqNet recognition model outperformed manual removal of ocular and cardiac artifacts. This improvement can be attributed to two key limitations of manual artifact removal: (i) subjective recognition of artifacts is prone to human error, and (ii) manual removal is performed across all epochs, thereby neglecting the real-time decomposition effects of artifacts and leading to residual errors. By contrast, the training and testing datasets of CA-SeqNet are derived from more objective correlation thresholds, which eliminates biases introduced by subjective judgment. Moreover, the model operates on individual epochs for artifact recognition and removal, further enhancing its performance. To facilitate reproducibility and enable broader application, we have made the source code for CA-SeqNet, including scripts for automatic recognition and removal of physiological artifacts, publicly available at: https://github.com/lewin-liyong/CA-SeqNet (accessed on 22 August 2025).

Importantly, OPM-MEG introduces unique challenges due to its flexible sensor placement and modular device design. Variability in channel arrangements across subjects or sessions can alter the projection patterns of artifacts, potentially complicating their recognition. However, these challenges are mitigated in the present framework: constructing datasets based on randomized dependence coefficient (RDC) correlations ensures the objective labeling of artifact components, regardless of the channel layout. Moreover, the introduction of the attention mechanism enables the model to adaptively emphasize discriminative artifact-related features, thereby enhancing the robustness of artifact recognition across varying sensor configurations. Together, these features demonstrate that CA-SeqNet accommodates OPM-specific experimental variability while maintaining reliable and automated artifact removal.

Nevertheless, several limitations should be noted. First, although second-generation QuSpin OPMs are capable of measuring orthogonal components, we employed a simplified radial-only configuration to reduce system complexity. Prior studies have validated the feasibility of such radial-only systems [42,43,44], yet the absence of tangential measurements remains a limitation in our study. Future work will incorporate multi-axis OPM data to further improve model accuracy and extend the applicability of the proposed framework across different experimental paradigms. Second, correlation analyses in this study did not account for potential time-lag effects between artifacts and reference signals, which may lead to underestimation of true correlations. Future studies may therefore investigate delayed correlation measures. Third, ICA decomposition was carried out under stationary assumptions, which may not hold in dynamic tasks such as mobile OPM-MEG experiments, where interference sources may move relative to the sensors. In such scenarios, standard ICA can be suboptimal, and optimizing ICA or adopting alternative decomposition schemes will be essential for future applications. Finally, while the VGG16_3_blocks-derived backbone yielded the best empirical performance, it may not represent the optimal architecture, and further exploration of backbone networks remains a promising direction.

In summary, this study demonstrates the feasibility of using magnetic reference signals in combination with a channel attention mechanism for automatic artifact recognition in OPM-MEG. The proposed CA-SeqNet framework provides reliable, reproducible, and fully automated artifact removal, thereby addressing key limitations of conventional approaches and offering a foundation for future extensions toward more complex OPM-MEG paradigms.

## Figures and Tables

**Table 1:** Statistics of sample number of time sequence signal datasets.

Statistical Items	Blink	Heartbeat	Non-Artifact
sample length	10s	10s	10s
sample points	10,000	10,000	10,000
number of subjects	12	12	12
number of samples before augmentation	480	480	1920
number of samples after augmentation	960	960	3840

**Table 2:** Basic concepts of classification problems.

Type	Definition
True Positive (TP)	Samples where the model prediction result is positive and the actual result is also positive, that is, the positive sample correctly predicted by the model
False Positive (FP)	Samples where the model prediction result is positive but the actual result is negative, that is, the model incorrectly predicts a positive negative sample
True Negative (TN)	Samples in which the model prediction result is negative and the actual result is also negative, that is, the negative sample correctly predicted by the model
False Negative (FN)	Samples where the model prediction result is negative and the actual result is positive, that is, the model incorrectly predicts a negative positive sample

**Table 3:** Performance comparison of related algorithm models.

Network Category	ACC	PR	RE	Macro-F1
LSTM	0.6667	0.2222	0.3333	0.2667
CA-LSTM	0.7161	0.4652	0.4844	0.4488
GRU	0.6658	0.3057	0.3342	0.2699
CA-GRU	0.7856	0.6265	0.6454	0.6242
AlexNet	0.8038	0.7065	0.6858	0.6953
CA-AlexNet	0.8186	0.7408	0.7049	0.7206
FasterRCNN1D	0.6667	0.2222	0.3333	0.2667
CA-FasterRCNN1D	0.8672	0.7976	0.7734	0.7848
DenseNet121	0.6667	0.2222	0.3333	0.2667
CA-DenseNet121	0.9479	0.9380	0.9154	0.9262
VGG16	0.6667	0.2222	0.3333	0.2667
CA-VGG16	0.6667	0.2222	0.3333	0.2667

**Table 4:** Performance comparison of VGG16_3_ blocks Ablation Study.

Ablation Study	ACC	PR	RE	Macro-F1
VGG16_3_blocks	0.7127	0.5737	0.5556	0.5632
CA-(VGG16_3_blocks)	0.9609	0.9506	0.9349	0.9426
CA-(VGG16_3_blocks_Maxpool1d)	0.8880	0.8314	0.8411	0.8359
CA-(VGG16_3_blocks_BatchNorm1d)	0.9488	0.9307	0.9223	0.9241
CA-SeqNet	0.9852	0.9836	0.9796	0.9815

**Table 5:** Comparison of performance indicators across categories.

Artifact Category	ACC	PR	RE	F1/Macro-F1
cardiac artifacts	99.47	98.87	96.88	97.86
blink artifacts	97.39	98.25	97.92	98.08
non-artifacts	98.70	97.96	99.09	98.52
total	98.52	98.36	97.96	98.15

**Table 6:** Comparison of signal-to-noise ration.

Types of Epochs	Subject1	Subject2	Subject3	Subject4
Raw	−0.72	3.18	1.86	1.94
Manually remove artifacts	4.72	9.19	8.52	8.38
CA-SeqNet automatically removes artifacts	8.11	11.28	9.15	9.13

## Data Availability

The data, aside from the data published in this manuscript, are not publicly available due to privacy restrictions. You can find the provided data at this link: https://zenodo.org/records/13997463 (accessed on 27 October 2024).
